# Soil-Transmitted Helminth Infection in Malaysia: Protocol for a Scoping Review

**DOI:** 10.2196/36077

**Published:** 2022-10-05

**Authors:** Muhammad Faiz Mohd Hisham, Fazila Haryati Ahmad, Hasmah Mohamed Haris, Noor Aliza Lodz, Norzawati Yoep, Eida Nurhadzira Muhammad, Rafidah Ali, Nor Asiah Muhamad

**Affiliations:** 1 Institute for Public Health National Institute of Health Shah Alam Malaysia; 2 Evidence Based Healthcare National Institute of Health Shah Alam Malaysia

**Keywords:** STH, soil-transmitted helminth, PRISMA-ScR, Malaysia, helminth, tropical disease

## Abstract

**Background:**

Soil-transmitted helminth (STH) infection is 1 of the 20 notable neglected tropical diseases according to the Centers for Disease Control and Prevention and World Health Organization. In 2010, it is estimated that 1.73 billion people are infected with STH globally, of which 70% of cases occur in Asia. To date, there is a dearth of published literature on the prevalence of STH infection throughout Malaysia.

**Objective:**

The objectives of this study are to review research activity on STH infection in Malaysia, to estimate the prevalence of STH infection among Malaysians, and to identify significant risk factors associated with the infection. This review aims to provide the current state of evidence pertaining to STH infections, focusing on the main areas, limitations, and biases of research and mapping out the morbidity distribution of the diseases and their causative agents, and to identify significant risk factors for preventive measures.

**Methods:**

We will conduct a scoping review based on the 6-stage structured framework developed by Arksey and O’Malley. A comprehensive search strategy focusing on STH infection will be executed using electronic databases (Scopus, PubMed, Web of Science, and Embase). A systematic approach for searching, screening, reviewing, and data extraction will be applied based on the PRISMA-ScR (Preferred Reporting Items for Systematic Reviews and Meta-Analyses extension for Scoping Reviews) guidelines. Mendeley software and Microsoft Excel will be used to manage the references and to remove duplicates. Relevant data from selected articles will be extracted using a standardized data extraction form.

**Results:**

A total of 164 potential manuscripts were retrieved. Data extraction is currently in progress and completion is expected by the end of 2022.

**Conclusions:**

Our scoping review will summarize the current state of research in this field and provide comprehensive information regarding STH infections in Malaysia for future reference.

**Trial Registration:**

National Medical Research Register NMRR-20-2889-54348; https://nmrr.gov.my/research-directory/e52ea778-d31c-4eb4-9163-a45bb3680bbf

**International Registered Report Identifier (IRRID):**

DERR1-10.2196/36077

## Introduction

Soil-transmitted helminth (STH) infection is among the most common diseases worldwide, primarily affecting those living in poor tropical and subtropical regions, especially households with inadequate sanitation facilities [1]. STH contamination can occur due to high soil moisture content in cramped living quarters, shared toilets, uncovered latrine pits, unhygienic practices, and pet (cat and dog) ownership, which increases the risk for zoonotic transmission [2]. STHs are nematodes including roundworms (*Ascaris lumbricoides*), whipworm (*Trichuris trichiura*), and anthropophilic hookworms (*Necator americanus* and *Ancylostoma duodenale*). STHs can infect humans through contact with parasitic eggs or larvae in soil [3]. 

According to the World Health Organization (WHO; 2005), STHs and schistosomes caused nearly 1.5 billion infections worldwide up to 2015. STH—namely, *Ascaris* spp, *Trichuris* spp, and hookworms—can affect physical and mental development in children, also contributing to poor nutritional status in the community [4,5]. Although it is known that hookworms can cause iron deficiency and protein malnutrition due to intestinal blood loss, STH infection does not necessarily cause death if early intervention and treatment are taken. STH infection can also cause anemia, where the intensity of hookworm infection is correlated to the depletion of host iron stores [6]. The prevalence of STH infections worldwide is overwhelming. According to Pullan et al (2014) [7], more than 50% of STH cases were recorded in South Asia and sub-Saharan Africa, with prevalence rates of *A lumbricoides*, *T trichiura,* and hookworm reported to be 819 million, 464 million, and 439 million, respectively. STH infection is very common in South Asia due to this region having tropical and moist climate areas, where these worms are endemic. It can also occur in several underdeveloped and developing countries in South Asia, which still do not have adequate clean water supply and do not have systematic sanitation infrastructure in some regions [8]. The highest prevalence of STH infections in South Asia was documented in India (21%) and China (18%), with the continent of Asia contributing to 67% of the global prevalence of STH infections [9]. Thirty-nine studies in India showed that *A lumbricoides* infection was the most prevalent parasite, with more than 50% prevalence reported in several states [10]. Conversely, the survey data of STH infection in China showed that the prevalence of STH infection in China considerably decreased from 2005 onward [11].

Although Malaysia is a developing country with rapid growth in socioeconomic and infrastructure in both urban and rural areas, the government is still grappling with the problem of STH infections, especially among very rural populations and indigenous communities. Many STH studies conducted in Malaysia focused on the indigenous people of Malaysia. Even though the government had built numerous resettlement areas for these indigenous tribes, they are still heavily dependent on the forest for their daily needs and sustenance, thereby retaining a high risk for intestinal parasitism [12]. A study by Sinniah et al (2014) [13] showed that STH infection was more common among those living in rural areas (32.3%), followed by urban squatters (20.6%) and those residing in flats or apartments (5.4%). The prevalence rate of STH infection among urban settlers, residents, and those living in flats showed a dramatic decrease, whereas STH infection prevalence in indigenous communities was over 90% previously (1970s) and is currently fluctuating below 70% (2000-2013) [13]. Another study revealed that the most prevalent types of STH in Malaysia are *T trichiura* (2.1%-98.2%), followed by *A lumbricoides* (4.6%-86.7%) and hookworm (0%-37%).

There are many recommendation documents published by the WHO to eliminate STH as a public health problem. The strategic plan for STH elimination included routine control activities in low-transmission areas, intensive control of STH infection in areas of high transmission (WHO 2001), and the delivery of anthelminthic treatment in school-age children to reduce worm loads (WHO 2012) [14]. In 1974, Malaysia launched a worm control program aimed at controlling STH infection [15]. The program targeted schoolchildren aged 7-15 years; a total of 1486 schools with more than 220,000 pupils were involved in this program. The national mass deworming program in Malaysia, which used a single dose of pyrantel pamoate once or twice per year, was discontinued in 1983 due to the drug’s low effectiveness against *Trichuris* and hookworm. Albendazole tablets are still given to children in some rural areas. The government also attempted to improve sanitation in rural households by providing pour-flush latrines and safe drinking water to diminish STH infection [16].

This review aims to provide the current state of evidence pertaining to STH infections, focusing on the main areas, limitations, and biases of research and mapping out the morbidity distribution of the diseases and their causative agents, and to identify significant risk factors for preventive measures.

## Methods

### Protocol Design

This study protocol is registered at the National Medical Research Register (NMRR-20-2889-54348) [17]. This scoping review will adhere to the 6-stage structured framework proposed by Arksey and O’Malley [18], which was further developed by Levac et al [19] and the Joanna Briggs Institute [20], where it is recommended that the review process be structured in at least 5 stages. These stages include (1) identifying the research question; (2) identifying relevant studies; (3) selecting studies; (4) charting the data; and (5) collating, summarizing, and reporting the results. Although stage 6 (consulting with relevant stakeholders) would be beneficial in terms of getting insights and updates on the present circumstances of STH infection in Malaysia, this scoping review will not include this stage due to time and budget constraints. However, experts with scoping review–writing experience and statisticians (for data analysis) may be consulted throughout the preparation of this scoping review. This protocol was not submitted to PROSPERO (International Prospective Register of Systematic Reviews), as they do not currently accept scoping review protocols. The report will follow the 22 items in the PRISMA-ScR (Preferred Reporting Items for Systematic Reviews and Meta-Analyses extension for Scoping Reviews) [21] guidelines. Clinical trial registration and ethics board approval are not needed since the review does not involve any human subjects.

### Stage 1: Identifying the Research Questions

An exploratory review of the literature on STH infection in Malaysia was conducted to refine the scope of this protocol and develop the research questions. Based on this review and through consultation with the research team, the following research questions were identified:

1. What types of research activity on STH infection have been carried out in Malaysia?
2. What is the prevalence of STH infection in Malaysia?
3. What are the significant risk factors associated with STH infection in Malaysia?

### Stage 2: Identifying Relevant Studies

A comprehensive search strategy will be executed by a team of investigators. Website sources will include published scientific journals, grey literature, and annual reports as below:

1. Electronic databases including PubMed, Scopus, Web of Science, and Embase
2. Relevant research websites such as ClinicialTrials.gov, the WHO, Global Atlas of Helminth Infections, Ministry of Health Malaysia, and Virtual Library Ministry of Health
3. Grey literature including website searches of universities, Google Scholar, and research institutes

A systematic approach to searching, screening, reviewing, and data extraction will be applied based on PRISMA-ScR guidelines. Titles, abstracts, and keywords will be examined for eligibility independently by 2 investigators. The proposed initial search strategy, keywords, and search terms for a search for related articles are attached. Medical Subject Headings (MeSH) terms were applied to assist the keyword search for different databases used (Multimedia Appendix 1). All selected search results will be downloaded and imported into Microsoft Word and Excel (Microsoft Corp) in duplicate; they will then be shared through Google Drive. Mendeley software and Microsoft Excel will be used to manage the references and to remove duplicates.

A hand search of the grey literature will be conducted through university visits and meetings with academics for further data retrieval and consultation, whenever relevant. The reference lists of publications by WHO-Western Pacific will be screened for additional sources of information.

### Stage 3: Study Selection

#### Overview

The study selection will be based on the objectives of this review, which are (1) to identify the trend of research activity (the extent and nature of study), (2) to estimate the prevalence of STH infection, and (3) to identify significant risk factors associated with STH infection in Malaysia. We will include all original articles, either observational (cohort study, case-control study, cross-sectional study, case report, ecological report, and descriptive report) or interventional (randomized and nonrandomized). The first level for the review process consists of the screening of titles and abstracts. Investigators will independently screen the title and abstract from all retrieved citations that meet the minimal inclusion criteria. Abstracts that do not meet the scope of the study will be excluded. The second level of screening will take place once relevant abstracts are selected. The full-text review will include any articles that are considered significant and applicable to the research question. Cohen κ statistic will be applied to determine interobserver agreement and ensure consistent application of the eligibility criteria for inclusion in the review [22]. The third investigator will review any full-text article assessment that does not meet perfect agreement (κ<1), and the discordance will be resolved through discussion until full consensus is reached.

#### Inclusion Criteria

The following principles will be used to determine the studies that meet the criteria:

1. Studies that present evidence that was published between 2000 and 2020.
2. Studies that present evidence that was carried out in Malaysia with the Malaysian population.
3. Studies that present evidence on STH infection incidents in Malaysia.
4. Studies published in the English language.

#### Exclusion Criteria

Studies with the following characteristics will be excluded:

1. Studies published before 2000.
2. Studies with no evidence on STH infection incidents in Malaysia.
3. Studies published in languages other than English.

### Stage 4: Charting the Data

The significant study characteristics from the articles will be extracted by a standardized data extraction framework using Google Sheets. It includes 7 sections that assist in data information extraction from the full review articles retrieved. Section 1 will provide standard bibliographical information (title, author, journal, year of publication, language, location of the study, sample size, and period of study), together with details pertaining to the specific STH involved in the study (parasite species of focus, predominant species, mixed infection, if a study was describing more than a single species of parasite, and the intensity of infection if mentioned). Sections 2 to 7 will describe the type of study, primary outcome, risk factors, treatment efficacy, laboratory investigation, and other valuable information, respectively. These sections will provide significant information about the study and facilitate data analysis (Multimedia Appendix 2). The data extraction framework will be distributed to all investigators through a link and can be easily accessed through email and mobile apps. Each investigator will be assigned articles in duplicate, and the results of the data extraction will be cross-checked with other investigators in the research team to ensure data extraction accuracy. Any aberrant findings and disagreements will be further discussed to ensure consistency and achieve consensus between investigators. A thorough discussion will be conducted whenever any questions or uncertainties arise throughout the whole data extraction process.

### Stage 5: Collating, Summarizing, and Reporting the Results

Results will be retrieved and downloaded through a spreadsheet generated using Google Sheets. All relevant information will be collated into its appropriate category and will be reported according to the selection criteria. The characteristics of the outcome from the selected articles will be described based on the types of interventions, study design, settings, tools used, and the outcomes of each study. The findings of this study will summarize all data and information from the relevant articles and emphasize the scope of STH infection in Malaysia. Topics and areas that have been under-studied and may require further attention might be identified and will be highlighted in this study.

### Ethical Considerations

Since the scoping review analysis seeks to synthesize information from publicly accessible publications and no primary data will be collected, formal ethical approval regarding dissemination activities is not necessary for this study.

## Results

The search was performed in December 2021, in the abovementioned electronic databases; a total of 164 results were retrieved. Data extraction from all potential manuscripts will be completed by the end of 2022. Data will be summarized descriptively in tabular form including types of interventions, study design, settings, tools used, and the outcomes of each study.

## Discussion

### Overview

Many publications focus on the prevalence and distribution of STH infections among the indigenous population in Malaysia based on sociodemographic characteristics. There are limited publications that specify the general population, laboratory investigation, and treatment efficacy. Those studies highlighted a single issue and were not as collaborative. There were also Knowledge, Attitude, and Practice studies that emphasized risk factors and disease prevention measures; however, those studies will not be selected for data collection in this scoping review. To ensure the report’s quality and reliability, only significant findings with *P*≤.05 will be included in this study. 

This scoping review will determine the types of research activities that have been carried out in Malaysia, whether epidemiological, clinical, treatment efficacy, preventive measures, or others, where related to the research topic. It will include all studies published between 2000 and 2020, as this time frame will provide adequate data to compare and summarize. We would like to provide further evidence on the prevalence of STH in terms of the parasite species that predominately cause the infection and the intensity of the infection. Prevalence figures provided by selected studies were calculated, considering each study’s sample size. Prevalence maps will be produced based on the geographical coordinates of the studies’ sites. Finally, we will present the significant risk factors that contribute to STH infection and discuss prevention measures taken by considering the government and private sector’s involvement toward curbing this issue. We hope that the findings of this scoping review will provide information for policy makers and strengthen policy guidelines to eradicate STH infection, as well as for researchers to further study and investigate any STH-related issue in Malaysia. 

### Dissemination

An article detailing the scoping review findings will be submitted to a scientific journal for publication and will be presented at relevant meetings and conferences, as well as for continuous medical education at the departmental level. The scoping review results are expected to provide a comprehensive overview of the available evidence on the prevalence of STH infection in Malaysia and to highlight areas of controversy or where evidence is lacking. It will also offer essential information to policy makers and health practitioners involved in designing, funding, and delivering evidence-based and effective strategies to prevent STH infection. The findings will also be disseminated as part of future seminars and workshops.

## Appendix

Multimedia Appendix 1 Table S1.

Multimedia Appendix 2 Table S2.

## Abbreviations

MeSH: Medical Subject Headings
PRISMA-ScR: Preferred Reporting Items for Systematic Reviews and Meta-Analyses extension for Scoping Reviews
STH: soil-transmitted helminth
WHO: World Health Organization
