# Supplementary material for: Soil-Transmitted Helminth Infection in Malaysia: Protocol for a Scoping Review
Source: JMIR Res Protoc. 2022 Oct 5;11(10):e36077. doi: 10.2196/36077 (PMC9582912; doi:10.2196/36077)
Supplement: Multimedia Appendix 1 [file resprot_v11i10e36077_app1.pdf]

Multimedia Appendix 1

Table S1 : Keyword Search

Keyword search	Search engine used	Number of publications retrieved
**(((helminth OR helminthiasis OR nematode OR "intestinal worm" OR "round word" OR "whip worm" OR "hook worm" OR "thread worm" OR "Ascaris lumbricoides" OR "Trichuris trichuria" OR "Ancylostoma duodenale" OR "Necator americanus" OR "Strongyloides stercoralis" OR ascariasis OR trichuriasis OR ancyclostomiasis[MeSH Terms]) AND ("soil-transmission helminth" OR "soil-transmitted helminth" OR "contaminated soil" OR STH[MeSH Terms])) AND (adult OR children OR "orang asli" OR indigenous OR tribe OR aborigine OR aboriginal OR native OR primitive OR "school-aged children" OR man OR woman OR "preschool children" OR population OR public OR patient[All Fields])) AND (Malaysia OR Malaysian[All fields]).**	PubMed<br>Scopus<br>Web of Science<br>Embase	38<br>65<br>7<br>53
