# Supplementary material for: Soil-Transmitted Helminth Infection in Malaysia: Protocol for a Scoping Review
Source: JMIR Res Protoc. 2022 Oct 5;11(10):e36077. doi: 10.2196/36077 (PMC9582912; doi:10.2196/36077)
Supplement: Multimedia Appendix 2 [file resprot_v11i10e36077_app2.pdf]

Multimedia Appendix 2

Table S2 : Data extraction framework

Category	Description
**Section 1 : Epidemiology of Study**	
**Title**	
**Author**	
**Journal**	
**Year published**	
**Language**	
**Location**	
**Sample size**	
**Period of study**	
**Population**	Specify Malaysian population (adult, children, elders)
**Study setting**	Specify the type of study setting (community-based, healthcare based, specific population-based)
**Parasite species of focus**	Specify types of helminths and infection in the study
**Predominant species**	
**Mixed infection**	
**Intensity of infection**	
**Section 2 : Type of Study**	Specify type of study (observational or interventional)
**Section 3 : Primary Outcome**	Specify the main outcome from the study (prevalence only, prevalence and risk factors, prevalence and pathological association and others)
**Section 4 : Risk Factors**	Specify the number of relevant data set and associated risk factors for STH infection
**Section 5 : Treatment Efficacy**	Specify the number of relevant data set, treatment platform, drugs and dosages and cure rate
**Section 6: Laboratory Investigation**	Specify the number of relevant data set and method used in laboratory investigation
**Section 7 : Other**	Any other significant findings
